# Natural Deep Eutectic Solvents for Simultaneous Extraction of Multi-Bioactive Components from Jinqi Jiangtang Preparations

**DOI:** 10.3390/pharmaceutics11010018

**Published:** 2019-01-04

**Authors:** Lele Yang, Ling Li, Hao Hu, Jianbo Wan, Peng Li

**Affiliations:** 1 State Key Laboratory of Quality Research in Chinese Medicine, Institute of Chinese Medical Sciences, University of Macau, Macau 999078, China; yb67542@connect.um.edu.mo (L.Y.); haohu@um.edu.mo (H.H.); jbwan@um.edu.mo (J.W.); 2 Bioengineering College, Xihua University, Chengdu 610000, China; lily1188@126.com

**Keywords:** deep eutectic solvents, Jinqi Jiangtang, ultrasound assisted extraction, response surface methodology, bioactive components

## Abstract

Natural deep eutectic solvents (NADESs), composed of natural primary metabolites, are now widely used as green and sustainable extraction solvents of bioactive components. In the present study, various NADESs were prepared to extract multi-components from different preparations of an herbal formula (Chinese name: Jinqi Jiangtang, JQJT) using ultrasound-assisted extraction (UAE). Results showed that most prepared NADESs provided more effective extraction of phenolic acids and alkaloids from JQJT preparations than conventional solvents. Among the tested NADESs, the solvent formed by choline chloride and laevulinic acid was selected to optimize the operational parameters using response surface methodology. The optimized extraction method was successfully applied to extract six major components in four commercial JQJT products, and quantification analysis was performed by the validated high-performance liquid chromatography with ultraviolet detection (HPLC-UV) method. The quantitative results indicated that preparations from different manufacturers showed different chemical profiles. In conclusion, NADESs-based UAE shows considerable potential as an efficient and green method for extraction of multi-bioactive components from commercial herbal preparations.

## 1. Introduction

The extraction of desired chemical compounds is the vital first step in quality assurance of pharmaceutical products [1]. Conventional organic solvents are commonly utilized in the extraction and purification of bioactive components [2]. However, most of these solvents are volatile, not environmentally friendly, and have toxic effects on the process operator. Moreover, they are not capable of efficient simultaneous extraction of both non-polar and polar components. Consequently, there is a great need to develop new extraction solvents that could preserve the environment and reduce the negative influence on human, and more importantly, allow for more efficient sample extraction in the analysis of pharmaceutical drugs [3].

The negligible toxicity and ionic liquid-like advantages, e.g., low volatility, high solubility, and high stability, allow deep eutectic solvents to be efficient green substitutes for conventional organic solvents [4]. Recently, a new type of deep eutectic solvent, known natural deep eutectic solvents (NADESs) have garnered great interest as greener and more efficient alternatives to conventional organic solvents. NADESs are commonly composed of two or more naturally occurring, non-toxic, and inexpensive compounds, which are bounded together through hydrogen bonding [4]. Due to their non-toxicity and the ability to dissolve both non-polar and polar compounds, NADESs have been considered as efficient and safe solvents to extract DNA, protein, and purify fuels [5,6,7]. NADESs have also been used in other hot and emerging fields of science, such as organometallics, organo-, metal-, and bio-catalysis [8,9,10,11,12,13], as well as in energy technologies [14] with unexpected findings. More recently, they have been successfully applied to extract diverse types of natural active compounds from botanicals, such as flavonoid [15], phenolic compounds [16], alkaloids [17], and so on. However, there have been no reports using NADESs as extraction media for simultaneous extraction of multi-bioactive compounds from herbal formulas, especially commercial available herbal preparations.

Jinqi Jiangtang (JQJT), an anti-diabetic patent herbal formula, consists of three herbal medicines including *Coptis chinensis*, *Astragalus membranaceus*, and *Lonicera japonica*, which have been widely used in clinical practice to prevent and treat type 2 diabetes [18]. Alkaloids and phenolic acids are two major bioactive components of JQJT with different polarities [19]. Due to the low water solubility of many active ingredients, various organic solvents have been used to extract chemicals from JQJT, which commonly consume a large amount of solvents. Thus, studies are required to explore the application of NADESs as green solvents in the extraction of multi-compounds from JQJT preparations.

In this study, we selected herbal formula JQJT to evaluate the efficiency of different NADESs on the extraction of multi-bioactive components with diverse polarity. After initial screening, the operation condition of NADES with high extraction efficiency was optimized using response surface methodology (RSM) in terms of water content, solid (sample powder)-liquid (solvent) ratio and ultrasonic irradiation time. The optimized NADESs extraction method was further applied to quantitative analysis of multi-bioactive components from various commercial preparations of JQJT (e.g., granule, tablet, and capsule).

## 2. Methods and Materials 

### 2.1. Materials 

Chemicals for preparation of NADESs including L-proline (≥99.0%) (Pro), choline chloride (≥ 98.0%) (ChCl), laevulinic acid (≥99.0%) (La), D-(+)-glucose (≥99.5%) (Glu), glycerol (≥99.0%) (Gly), and DL-malic acid (≥99.0%) (Ma) were obtained from Aladdin Reagent Company (Shanghai, China). The reference standards of chlorogenic acid (≥98.0%), neochlorogenic acid (≥98.0%), isochlorogenic acid B (≥98.0%), coptisine (≥98.0%), groenlandicine (≥98.0%) and berberine (≥98.0%) were purchased from Chengdu Must Biotechnology Co., Ltd. (Chengdu, China). High-performance liquid chromatography (HPLC) grade acetonitrile was obtained from Merck (Darmstadt, Germany). Ultrapure water was produced by a Milli-Q water purification system (Millipore, Milford, MA, USA). All other chemicals used in this study were of analytical grade. Four commercial preparations of JQJT were bought from pharmaceutical markets: JQJT-1, JQJT granule from Zhongjing Wanxi Pharmaceutical Co., Ltd. (Zhengzhou, China); JQJT-2, JQJT tablet from Zhongxin Pharmaceutical Co. Ltd. (Tianjin, China); JQJT-3, JQJT capsule from Tianyitang Pharmaceutical Co., Ltd. (Zhejiang, China); JQJT-4, JQJT capsule from Xianzhen Pharmaceutical Co., Ltd. (Liaoning, China).

### 2.2. Apparatus and Conditions 

The HPLC with ultraviolet detection (HPLC-UV) analysis was performed using an Agilent 1200 HPLC system (Agilent Technologies, Santa Clara, CA, USA), equipped with an on-line degasser, a binary solvent delivery system, an auto-sampler, a column temperature controller and a diode array detector (DAD) on a C_18_ column (4.6 mm × 250 mm, 5 µm, Shiseido Co., Ltd., Tokyo, Japan). The mobile phase consisted of water with 2% H_3_PO_4_ (A) and acetonitrile (B). The gradient program was as follows: 10–15% B (0–10 min), 15–25% B (10–15 min), 25% B (15–30 min) at a flow rate of 1.0 mL/min. The injection volume was 10 µL. The re-equilibration time after a gradient run was 8 min, and chromatograms were recorded at 335 nm. The data acquisition and analysis were performed using Agilent ChemStation software (Agilent Technologies). 

The HPLC-UV method employed in this work was validated in terms of linearity, precision, repeatability, stability, and recovery. Linearity was determined by at least six different concentrations in triplicate. The peak areas (*y*) and the corresponding concentration (*x*, μg/mL) were used to construct the calibration curves. The intra- and inter-day precision were determined by the analysis of quality control samples at three levels (low, medium, and high), during a single day and on three subsequent days, respectively. Five different working solutions were prepared to confirm the repeatability. The obtained relative standard deviations (RSDs) was used to evaluate the precision and repeatability. To evaluate the stability, samples were stored at room temperature for 0 h, 6 h, 12 h, 24 h, and then the RSDs of peak areas of all analytes were calculated. The recovery was determined by spiking accurate amounts of six compounds to approximate 100 mg JQJT preparations using the equation: recovery (%) = (found amount − original amount)/spiked amount × 100.

### 2.3. Sample Extraction Procedure 

An accurately weighed powder (8 mg) of each JQJT product was extracted with 1 mL of different solvents in a 1.5 mL microtube, and then ultra-sounded for 30 min. The extraction mixtures were centrifuged at 12,000 *g* for 10 min. After diluted ten times with water, the suspension was filtered by 0.45 µm filters before HPLC analysis. Each experiment was performed in triplicate. 

### 2.4. Preparation of NADESs

NADESs were prepared by heating as previous described [17]. Briefly, the mixtures of two or three compounds for the preparation of NADESs at a proper molar ratio were added to a reaction flask. The mixtures were heated at 80 °C with magnetic agitation until a homogeneous liquid was formed (Table 1). 

### 2.5. Experimental Design and Statistical Analysis

The Box-Behnken Design (BBD), a type of RSM, was performed using the Design-Expert Ver 8.0.6 (Stat-Ease Inc., Minneapolis, MN, USA). Three independent variables including water content (%) (A), solid (sample powder)-liquid (solvent) ratio (B) and ultrasonic irradiation time (C) were investigated at three levels (−1, 0, 1), as shown in Table 2. Data analysis were accomplished by Graph PadPrism 6.02 for Windows (Graph Pad Software, San Diego, CA, USA). The *p* values < 0.05 were considered significant.

## 3. Results and Discussion

### 3.1. Selection of Compounds for Evaluating the Extraction Efficiency with Different Solvents

The chosen compounds should comprehensively reflect the extraction efficiency of different solvents based on diverse chemical structures, different concentration levels, wide polarity range, and therapeutic activity [16]. Herbal medicines, especially multi-herbal formulas, contain complex constituents taking effect via a multi-target additive, synergistic, and/or competitive mode [20]. Chemical investigations into JQJT preparations revealed several kinds of active ingredients related to the treatment of type 2 diabetes and associated complications, including alkaloids, phenolic acids, and flavonoids [21]. According to current Chinese Pharmacopoeia (2015), berberine is a major quality control marker for JQJT preparations. Moreover, previous studies have demonstrated the beneficial effects of alkaloids and phenolic acids, such as chlorogenic acid, neochlorogenic acid, isochlorogenic acid, coptisine and berberine, in the management of diabetes [21]. 

**Figure 1:**
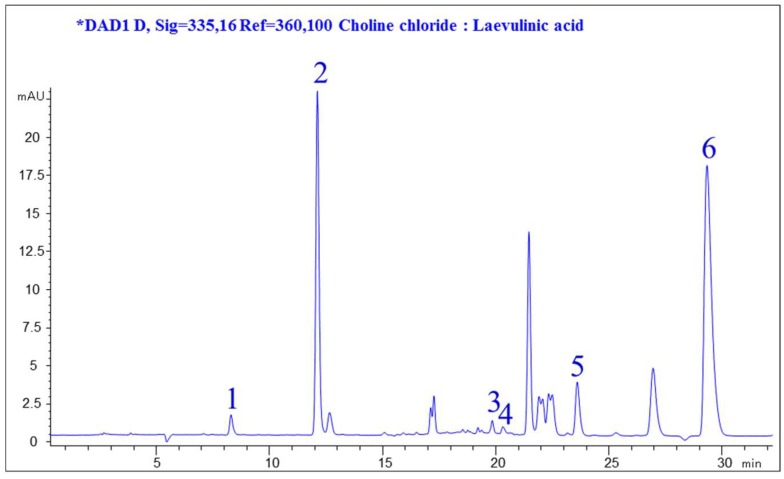
Representative HPLC chromatogram of Jinqi Jiangtang (JQJT) extract recorded at 335 nm. (1, Neochlorogenic acid, NeoCha; 2, Chlorogenic acid, Cha; 3, Groenlandicine, Gro; 4, Isochlorogenic acid B, IsoCha; 5, Coptisine, Cop; 6, Berberine, Ber).

As shown in Figure 1, the extracted compounds with a wide range of polarity were well separated and covered most of the practical HPLC elution range. Since the interference was caused by background, minor compounds, and other co-elutes ingredients, six peaks were chosen for subsequent comparison of the extraction efficiency of different solvents. Among these chromatographic peaks (Figure 1), the compounds were identified by comparing the retention time with that of standard references, which represented major different groups of anti-diabetic ingredients within a wide polarity range in JQJT extract. Consequently, the above-mentioned reference was selected to evaluate the extraction efficiency of different NADESs.

### 3.2. Comparison of Extractability of Multi-Compounds from JQJT with Different Solvents

The interaction of hydrogen bonding between the hydrogen bond acceptors (such as choline chloride) and the hydrogen-bond donor (such as glycerol) is the main force required to produce NADESs. The tunable features of NADESs greatly influences their extraction efficiency of multi-compounds of different polarity [22]. Choline chloride and proline are two most widely used components for preparation of NADESs [22,23]. In combination with appropriate primary metabolites, such as glucose, organic acids (e.g., malic acid), or polyols (e.g., glycerol), choline chloride and proline are capable of forming NADESs [24]. In addition, several reports have demonstrated the potential of proline/choline chloride-based NADESs for the extraction of compounds from herbal medicines [23,24]. In this study, nine proline/choline chloride-based NADESs with different composition, viscosity, and polarity were prepared to perform the extraction of different compounds from JQJT preparations. 

A key factor of using NADESs as extraction solvents is viscosity, and previous studies reported that the high viscosity would reduce their extraction efficiency [16]. In the present study, therefore, a certain volume of water (25%, *v*/*v*) was added to NADESs to adjust the solvent viscosity before subsequent extraction [22]. Methanol containing various amounts of water is commonly used for extraction of herbal medicines and related preparations in Chinese pharmacopoeia. Thus, an aqueous solution of 70% methanol was used as a common extraction solvent to perform extractability comparison with that of different NADESs, while water was employed as a sustainable solvent. Ultrasound-assisted extraction (UAE) was selected to perform initial screening due to its effective, simple, and fast features in the extraction of natural product [25]. Generally, 8 mg of the JQJT preparations was mixed with 1 mL of different solvents, including 70% methanol, 75% NADESs, and water, respectively, and then the mixtures were extracted by UAE for 30 min. 

**Figure 2:**
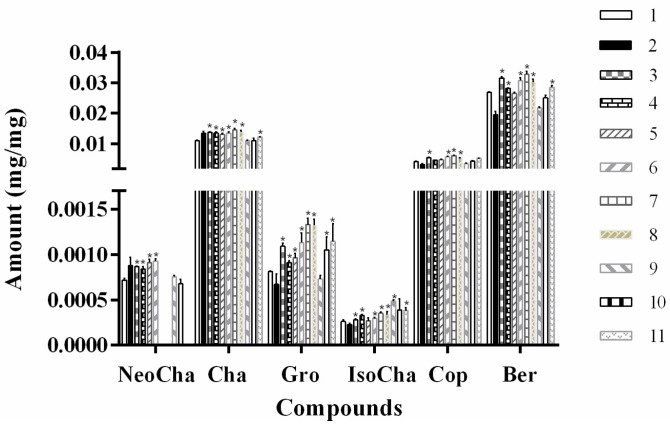
Extraction yields (mg of compounds per mg of JQJT powders) of tested NADESs for different compounds. Numbers represent the extract solvents in Table 1.

The HPLC-UV method was used for quantitative determination of the phenolic acid and alkaloids in various solvent extracts. Results showed that the extraction yields (mg/g) of the compounds obtained from different tested solvents varied considerably (Figure 2). The yield of these components in water and methanol was closely related to their polarity, which was indicated by the elution order in HPLC analysis. The 70% methanol extracts gave a higher dissolving capacity of less polar compounds berberine and coptisine, while water was more suitable for extraction of relative polar compounds. All the investigated NADESs exhibited high extraction efficient for most of the target compounds (Figure 2), which was consistent with previous reports that the extraction efficiency of NADESs for phenolic acids was superior to that of methanol [16,26]. No significant differences were observed in the extraction efficiency of NADESs and water for neochlorogenic acid and chlorogenic acid. Duan et al. [17] showed that natural-occurring alkaloids could be highly extractable in laevulinic acid-based NADESs. Similarly, when using laevulinic acid-based NADESs (ChCl-La and Pro-La), the yields of alkaloids, including coptisine, groenlandicine, and berberine were significantly increased. 

A variety of applications of liquid choline chloride mixtures have been proposed in various fields, such as trace metal dissolution [27], extraction of biodiesel [28], and purification of chemicals [29]. Work by Bi et al. showed the potential of NADESs containing choline chloride and different alcohols to extract and determinate of bioactive compounds (myricetin and amentoflavone) [29]. In addition, ball mill-assisted NADESs-based extraction has proven to be a fast and environmentally friendly method for the extraction of slightly water-soluble natural products from *Salvia miltiorrhiza bunge* [30]. Consistent with previous observations, choline chloride-based NADESs (ChCl-La, ChCl-Gly, ChCl-Glu, ChCl-Ma and ChCl-Pro) used in the present study exhibited rather high extraction efficiency compared with the control solvent (70% methanol). Among the investigated NADESs, ChCl-La, ChCl-Glu-Ma, ChCl-Pro, and Pro-La provided the highest extraction yield of the chosen compounds from JQJT preparations. However, ChCl-Pro and Pro-La had an influence on the detection of NeoCha. ChCl-La was much easier to prepared compared to that of ChCl-Glu-Ma, ChCl-Pro, and Pro-La. Given the above considerations, ChCl-La of 1:2 ratio was selected as the ideal extract solvent to optimize the operational parameters in the following procedure.

### 3.3. Optimization of the NADESs Extraction Condition

The operational conditions of extraction, including the NADESs/water ratio, solid−liquid ratio and extraction time, greatly affect the extraction capacity of target compounds. RSM is a less time-consuming and laborious statistical technique for optimization of the multiple parameters and their interactions [31]. BBD, a more efficient and easier RSM, was used to evaluate the extraction parameters in this study [25]. To promote the applications and development of NADESs as a green extraction solvent, many studies on the optimization of extraction parameters have been performed by using RSM [22,23]. Based on preliminary observations (data not shown), an experimental three-factor and three-level BBD-RSM was employed to optimize the following variables: water content (A, 25%–75%), solid/liquid ratio (B, 8–24 mg/mL), and extraction time (C, 20–60 min) (Table 2). The yield of six selected compounds from JQJT preparations was considered as the response of BBD-RSM method. 

The variables and response were analyzed and resulted quadratic multiple regression models for tested compound was built. Table 3 summarizes the results of variance (ANOVA) for the quadratic predictive model. The models were expressed as second order polynomial quadratic equations for the extraction yield (Y) and coded factors (A, B, and C) as follows:

$$\begin{array}{l} Y=0.059149+6.12657E^{-004}\times A-7.61268E^{-004}\times B\\ -3.52148E^{-004}\times C+1.35367E^{-005}\times A\times B+3.28315E^{-006}\\ \times A\times C-5.63531E^{-006}\times B\times C-1.04228E^{-005}\times A^{2}\\ +3.14353E^{-006}\times B^{2}+4.18853E^{-006}\times C^{2} \end{array}$$

**Figure 3:**
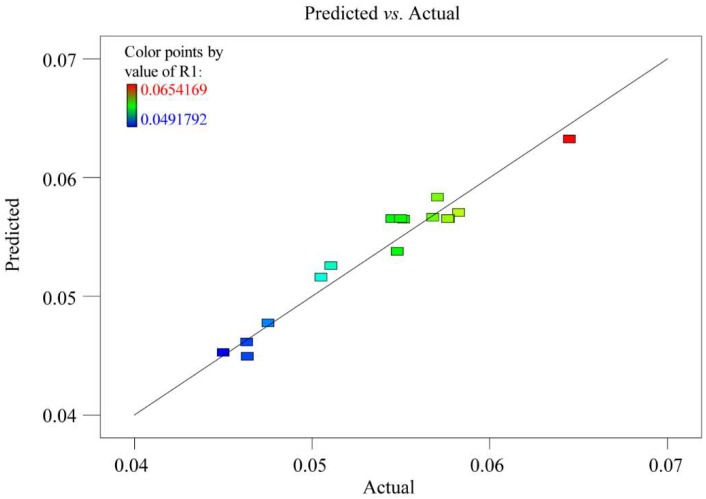
Design-expert plot: Predicted vs. actual values plot for the extraction yield of six selected compounds (R1).

The square of the correlation coefficient (*R*^2^) and the lack-of-fit was employed to evaluate the model quality. The value of *R*^2^ in this model was 0.95, which suggested that the experiment values corresponded well with the predicted yields. The measured F value of 1.71 and *p* value of 0.30 for lack-of-fit were not significant, indicating the obtained model could perfectly explain all data. In addition, the variation (CV) with a relatively low value of 2.41 indicated a very low degree of deviation and a good deal of reliability of the experimental values. The model satisfactoriness was further judged by diagnostic plots. The predicted versus actual values plots of the extraction yield was presented in Figure 3. The plot indicated an adequate agreement between the predicted values obtained from the models and the observed ones. The *p* value was used to determine the significance of each coefficient. As observed in Table 3, the obtained model was significant with very small *p* value (*p* = 0.001), which demonstrated that this model could be utilized to evaluate the independent variables and their relationship.

**Figure 4:**
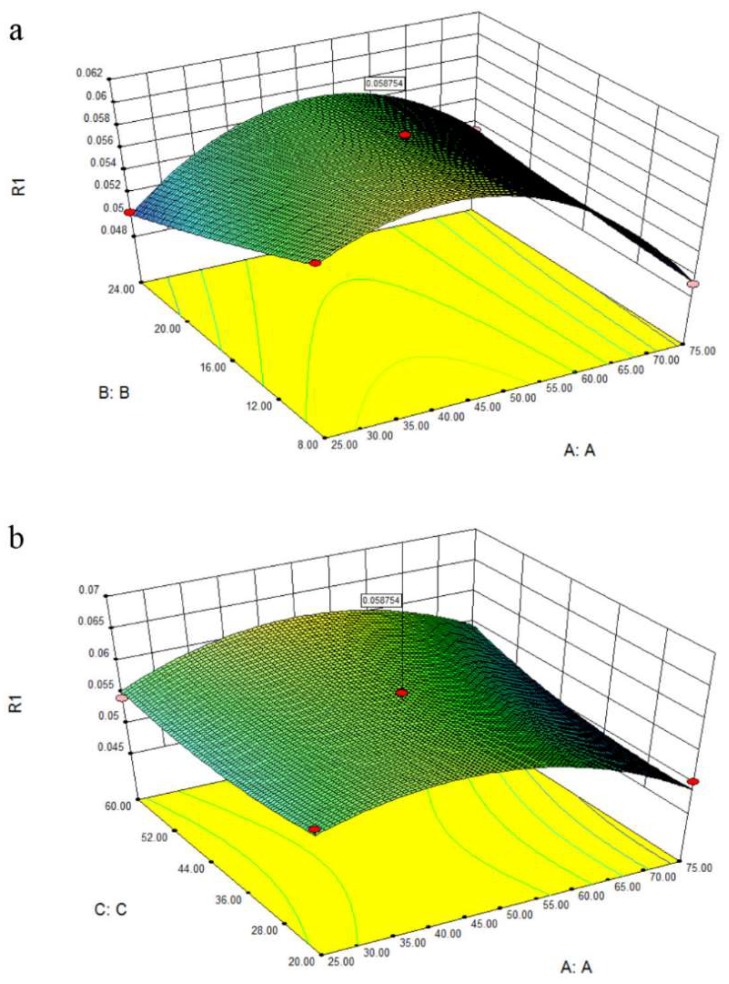
The three-dimensional response surface of obtained model.

The three-dimensional response surface in Figure 4 provided a graphic relationship between response and tested variables. The response surface plot illustrated in Figure 4a demonstrated the effect of water content and solid/solvent ratio on extraction yield, with time maintained at 20 min. It could be observed that the extraction ability was significantly influenced by water content and solvent/solid ratio. The extraction yield of target compounds increased with the increase of water content, and reached the maximum at the water content of 50%. Then the extraction efficiency rapidly decreased when the water content was higher than 50%. This phenomenon might be correlated with the decreased interaction between NADESs and target compounds, and the disappearance of hydrogen bonding with more than 50% water dilution [32,33]. Figure 4b shows the three-dimensional plot at varying extraction time and solid/liquid ratio to JQJT preparations at fixed water content (0 level). The extraction time had positive effects on extraction efficiency, and the extractability increased with lower solid/liquid ratio. The optimal extraction conditions for target compounds were presented as follows: water content of 50%, extraction time of 60 min, and solid/liquid of 8 mg/mL. 

### 3.4. Validation of the HPLC-UV Method

Method validation is an essential requirement for the quantitative analysis of pharmaceutical products. The developed HPLC-UV method was validated by determination of linearity, precision, stability, and recovery, and the validation results were shown in Table 4 and Table 5. The correlation coefficient was above 0.999 for all analytes, indicating the good linear regression in tested concentration range. With a signal-to-noise (S/N) ratio of 3 and 10, the limits of detection (LOD) and quantitation (LOQ) for these compounds were in a range of 0.02–0.1 μg/mL and 0.1–0.2 μg/mL detected in UV at 335 nm, respectively (Table 4). The intra-day and inter-day precision (RSD%) were in the range of 0.29% to 7.15% and 1.19% to 8.41% (Table 5), demonstrating the good stability and repeatability of the established method. The results of stability assay were less than 5.34%, indicating that all analytes were stable within 24 h. Moreover, the extraction recoveries of 6 analyses ranged from 98.50% to 103.15% with RSDs less than 5.14%. Therefore, the present HPLC-UV method was sensitive and reliable to simultaneously quantify multi-compounds in JQJT preparations. 

### 3.5. Analysis of Four Commercial JQJT Preparations

**Figure 5:**
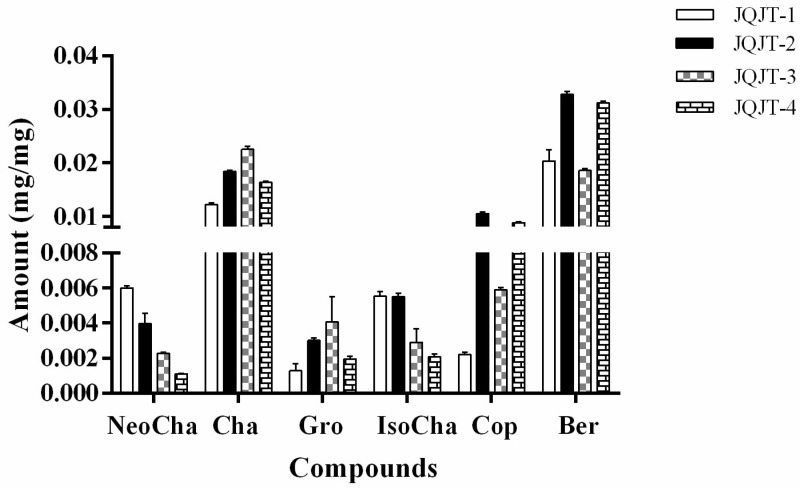
Quantitative analytical results of six components in different JQJT preparations.

The established HPLC-UV method was applied for quality evaluation of JQJT preparations through simultaneous quantitative determination of six selected bioactive compounds. The JQJT preparations were extracted by using the newly proposed NADESs-based UAE method. The quantitative analytical results of JQJT samples shown in Figure 5 indicated that the concentration of each compound present in individual preparations varied greatly, and the content of each analyte in different preparations was also different.

Berberine and chlorogenic acid are the most abundant alkaloids and phenolic acids, respectively. The levels of berberine, a bioactive and marker compound for quality control of JQJT formula, ranged from 0.019 mg/mg to 0.033 mg/mg. The content of chlorogenic acid was in the range of 0.012 mg/mg to 0.023 mg/mg. JQJT preparations were manufactured and sold widely as a Chinese patent drug. The variability of tested compounds content in different preparations was most likely due to different dosage forms and doses of herbal products. The different chemical profiles of JQJT preparations from four manufacturers indicated the necessity and urgency to establish a more comprehensive method for quality control of herbal medicines and related products. 

## 4. Conclusions

In this work, a green and efficient NADESs-based UAE technique was proposed and applied to simultaneously extract multi-bioactive components from JQJT products. This was the first attempt to extract alkaloids and phenolic acids from herbal preparations. It was observed that ChCl-La (molar ratio of 1:2) proved to be more effective than the methanol–water solution for simultaneous extraction of multi-compounds with a wide range of polarity. Subsequent optimization results using RSM demonstrated that the extraction efficiency was significantly influenced by water content and solid/liquid ratio. Moreover, the concentrations of the investigated chemicals in JQJT products from different manufacturers varied greatly, which is a bottleneck in herbal medicines modernization and globalization. This study may extend the applications of NADESs as promising green and efficient solvents for extraction of interested compounds in pharmaceutical products.

## Figures and Tables

**Table 1:** List of natural deep eutectic solvents (NADESs) for initial screening.

No.	Solvents	Mole Ratio
1	70% MeOH	
2	Water	
3	ChCl-La	1:2
4	ChCl-Gly	1:2
5	ChCl-Glu	1:1
6	ChCl-Glu-Ma	1:1:1
7	ChCl-Pro	1:2
8	Pro-La	1:2
9	Pro-Gly	1:1
10	Pro-Glu	1:1
11	Pro-Ma	1:1

**Table 2:** Investigated variables and levels for the Box-Behnken Design (BBD).

Variables	Unit	−1	0	1
water	%	25	50	75
solid/solvent	mg/mL	8	16	24
time	min	20	40	60

**Table 3:** ANOVA results of the obtained model for the extraction of 6 compounds.

Title	Sum of		Mean	*F*	*p*-Value	Significance
Source	Squares	df	Square	Value	Prob > F	
Model	2.96E-04	9	3.29E-05	14.18	0.001	significant
A-A	3.34E-05	1	3.34E-05	14.41	0.0068	
B-B	2.24E-05	1	2.24E-05	9.67	0.0171	
C-C	1.04E-05	1	1.04E-05	4.47	0.0722	
AB	2.93E-05	1	2.93E-05	12.65	0.0093	
AC	1.08E-05	1	1.08E-05	4.65	0.0679	
BC	3.25E-06	1	3.25E-06	1.4	0.2748	
A^2	1.79E-04	1	1.79E-04	77.1	<0.0001	
B^2	1.70E-07	1	1.70E-07	7.40E-02	0.7941	
C^2	1.18E-05	1	1.18E-05	5.1	0.0585	
Residual	1.62E-05	7	2.32E-06			
Lack of Fit	9.10E-06	3	3.03E-06	1.71	0.3029	not significant
Pure Error	7.12E-06	4	1.78E-06			
Cor Total	3.12E-04	16				
R²	0.95					
CV	2.41					

**Table 4:** Calibration curves, linear range, limits of detection (LODs) and quantifications (LOQs) of six compounds.

Compounds	Calibration Curve	Linear Range (μg/mL)	R²	LOD (μg/mL)	LOQ (μg/mL)
Neochlorogenic acid	y = 14280x − 4.4111	0.16–42	0.9999	0.024	0.1
Chlorogenic acid	y = 15806x − 4.5094	0.16–167	1	0.024	0.1
Groenlandicine	y = 7672x − 0.7485	0.5–142	1	0.1	0.2
Isochlorogenic acid B	y = 16377x − 23.675	0.16–167	0.9990	0.02	0.1
Coptisine	y = 6957.4x − 6.4411	0.16–167	0.9995	0.04	0.1
Berberine	y = 15899x − 15.411	0.16–167	0.9995	0.09	0.16

**Table 5:** Precisions, repeatability, stabilities and recovery of six compounds.

Compounds	Concentration (mg/mL)	Precision		Repeatability (*n* = 3)	Stability (*n* = 3)	Recovery (*n* = 3)
		Intra-Day (RSD%)	Inter-Day (RSD%)	RSD%	RSD%	Mean ± SD, %
Neochlorogenic acid	0.000425	0.82	1.19	4.82	2.09	100.44 ± 5.14
	0.00095	2.50	1.78			
	0.01	0.29	7.93			
Chlorogenic acid	0.000425	1.42	4.28	4.34	1.19	101.18 ± 3.14
	0.00095	2.45	2.00			
	0.01	0.55	7.78			
Groenlandicine	0.000425	6.30	4.28	4.38	1.59	100.25 ± 3.57
	0.00095	1.56	2.66			
	0.01	0.54	8.02			
Isochlorogenic acid B	0.0003	5.34	3.23	5.62	5.34	98.50 ± 3.03
	0.01	0.68	8.01			
	0.1	7.15	8.24			
Coptisine	0.000425	5.37	3.60	2.85	1.42	103.15 ± 0.29
	0.01	0.48	8.15			
	0.1	7.09	8.18			
Berberine	0.0008	0.51	5.32	4.01	1.68	102.06 ± 2.16
	0.01	1.01	8.41			
	0.1	5.81	7.79			
